# Tissue expression profiles unveil the gene interaction of hepatopancreas, eyestalk, and ovary in the precocious female Chinese mitten crab, *Eriocheir sinensis*

**DOI:** 10.1186/s12863-019-0716-1

**Published:** 2019-01-25

**Authors:** Xiaowen Chen, Jun Wang, Xin Hou, Wucheng Yue, Shu Huang, Chenghui Wang

**Affiliations:** 1 Key Laboratory of Freshwater Aquatic Genetic Resources, Ministry of Agriculture, Shanghai Ocean University, 999, Hucheng huan Road, Lingang New City, Shanghai, 201306 China; 2 National Demonstration Center for Experimental Fisheries Science Education (Shanghai Ocean University), 999, Hucheng huan Road, Lingang New City, Shanghai, 201306 China; 3 Shanghai Engineering Research Center of Aquaculture, 999, Hucheng huan Road, Lingang New City, Shanghai, 201306 China

**Keywords:** Transcriptome, Ovary development, Genetic network

## Abstract

**Background:**

Sexual precocity is a common biological phenomenon in animal species. A large number of precocity individuals were identified in Chinese mitten crab *Eriocheir sinensis*, which caused huge economic loss annually. However, the underlying genetic basis of precocity in *E. sinensis* remains unclear to date.

**Results:**

In this study, morphological and histological observation and comparative transcriptome analysis were conducted among different stages of precocious one-year-old and normal two-year-old sexually mature *E. sinensis*. The expression profiles of the ovary, hepatopancreas, and eyestalk tissues were presented and compared. Genes associated with lipid metabolic process, lipid transport, vitelline membrane formation, vitelline synthesis, and neuropeptide hormone-related genes were upregulated in the ovary, hepatopancreas, and eyestalk of precocious *E. sinensis*. Our results indicated that the eyestalk was involved in the neuroendocrine system providing neuropeptide hormones that may induce vitellogenesis in the hepatopancreas and further stimulate ovary development. The hepatopancreas is a site for energy storage and vitellogenin synthesis, and it may assist oogenesis through lipid transport in precocious *E. sinensis*.

**Conclusion:**

We provided not only an effective and convenient phenotype measurement method for the identification of potential precocious *E. sinensis* detection but also valuable genetic resources and novel insights into the molecular mechanism of precocity in *E. sinensis*. The genetic basis of precocity in *E. sinensis* is an integrated gene regulatory network of eyestalk, hepatopancreas, and ovary tissues.

**Electronic supplementary material:**

The online version of this article (10.1186/s12863-019-0716-1) contains supplementary material, which is available to authorized users.

## Background

Sexual precocity, which refers to the early maturity of the reproductive system (gonad) during puberty, is a natural phenomenon in most animal species, even humans [1–3]. This phenomenon can cause growth and development retardation, increased illness rate, and other associated physiological defects [1, 4]. Sexual precocity is a complex physiological process induced by extrinsic environmental factors and intrinsic genetic factors [2, 5]. The early development of gonads is considered a molecular response to environmental factors, such as hormones, nutrition, temperature, and disease [5]. However, the genetic mechanism underlying sexual precocity remains unclear to date.

In vertebrate, gonad development is regulated by the hypothalamus-pituitary-gonad axis (HPG). However, in invertebrate, the regulation of reproductive system is vague [6, 7]. The Chinese mitten crab *Eriocheir sinensis* is an economic crustacean widely cultured in China that suffers from severe precocious problems [8]. Huge economic losses in the *E. sinensis* aquaculture industry are caused by substantial proportions of precocious *E. sinensis* individuals every year [2, 9]. Environmental factors such as temperature, salinity, light, and stocking density induce sexual precocity in *E. sinensis*, however, the intrinsic molecular response to the stimulation of environmental factors is largely unknown in *E. sinensis* [10–12].

The X-organ-sinus gland complex neuroendocrine system in eyestalk functions similarly to the HPG axis in crustaceans [7, 13, 14]. Gonad inhibiting hormone (*GIH*), molt-inhibiting hormone (*MIH*), crustacean hyperglycaemic hormone (*CHH*), and neuropeptide F (*NPF*) genes/neuropeptides expressed and synthesized in eyestalk play essential roles in regulating the gonad development of *E. sinensis* [15]. Meanwhile, the hepatopancreas is an essential organ for energy metabolism, providing essential energy source for the gonad development of *E. sinensis* [16]. Studies have also indicated exogenous vitellogenin is synthesized in the hepatopancreas and transferred to the ovary during vitellogenesis process in *E. sinensis* [17]. However, how the environmental factors stimulate and activate the early gonad development and the specific biological function of eyestalk and hepatopancreas in regulating gonad development are largely unknown in precocious *E. sinensis*.

**Fig. 1:**
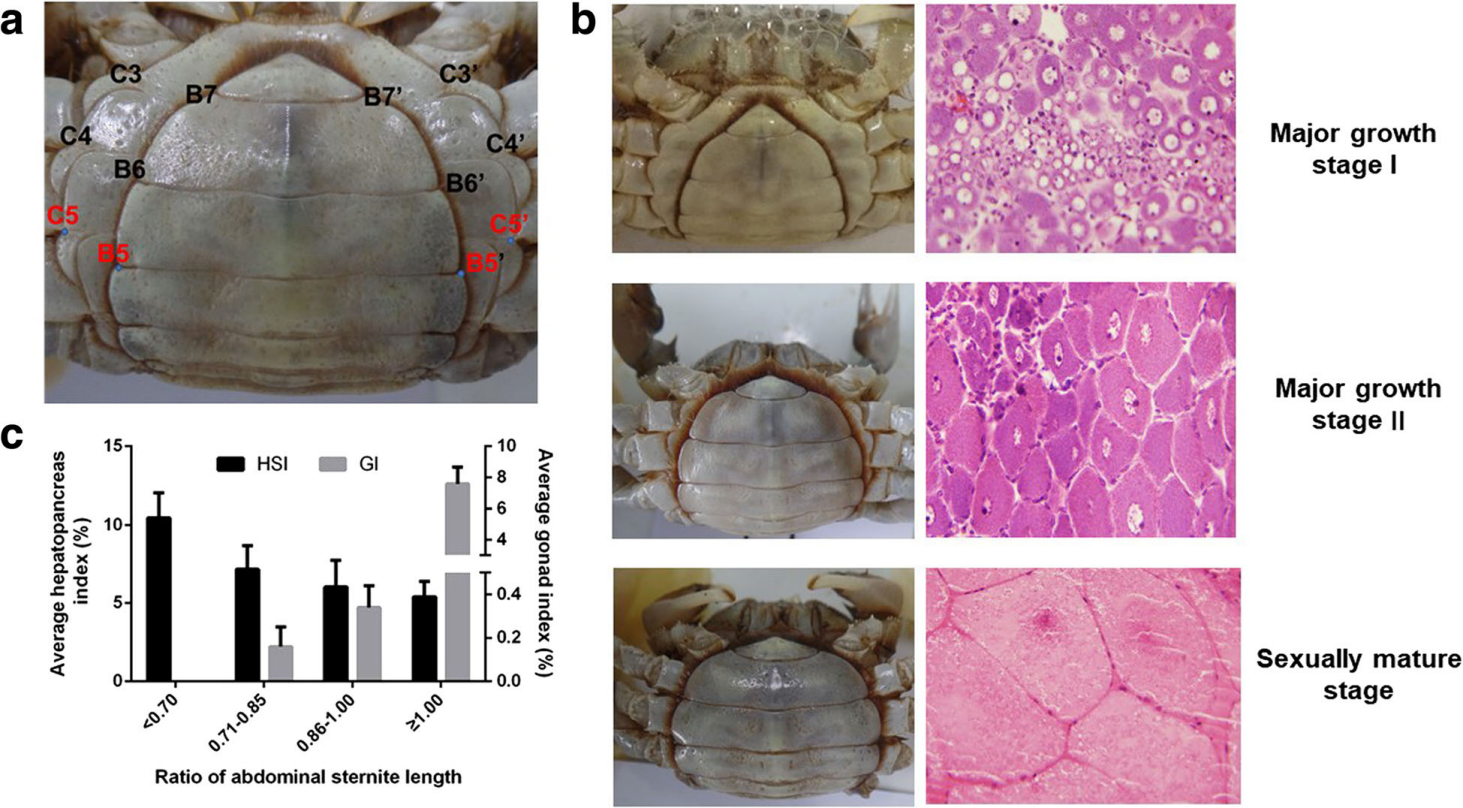
Phenotypic characters, histological observation, and hepatopancreas and gonad indexes among different ovary developmental stages of precocious *E.sinensis*. **a**. Shape of abdominal sternite of female *E.sinensis*. B5-B5’ indicates the outside length of the fifth abdominal sternite, and C5-C5’ indicates the inside length of the fifth abdominal sternite. **b**. Different shapes of abdominal sternite corresponding to different ovary developmental stages in female *E. sinensis*. The tissue-slices figure was photographed under 100× in LEIKA microscope. **c**. Average hepatopancreas and gonad indexes among different groups of precocious *E.sinensis.* HSI: hepatopancreas index, GI: gonad index

*E. sinensis* is a catadromous species with a life cycle of two years. Mating and spawning occur during winter in brackish water; fertilized eggs develop into larvae in spring; and then the larva will migrate to freshwater rivers/lakes and spend nearly two years with nearly 20 times molting before they reach sexual maturity [8, 18]. In general, the gonad development of *E. sinensis* initiates at the second year. As for precocious *E. sinensis*, the gonad starts to develop and reach complete sexual maturity in the first year [8]. Molting and growth are terminated in sexually mature precocious *E. sinensis* and these individuals are usually discarded because of their unworthiness in the aquaculture industry [9]. The most direct and accurate way to identify precocious *E. sinensis* is through histological observation, which is inconvenient during aquaculture for farmers. During the aquaculture process, experienced farmers identify precocious female *E. sinensis* individuals based on the shape of the abdominal sternite (Fig. 1a) [15, 19]. The female *E. sinensis* reach sexual maturity when the abdominal sternite completely covers the whole abdomen (Fig. 1a). Previous studies indicated the ratio of abdominal sternite length is a candidate phenotypic character to discriminate the precocious level in female *E. sinensis* (Fig. 1a) [19]. However, researches linking the abdominal sternite length to ovary developmental stages are limited.

In this study, potential precocious female *E. sinensis* individuals were collected based on phenotypic characters (ratio of abdominal sternite length) and then confirmed by histological observation. Comparative transcriptome analysis was conducted on the eyestalk, hepatopancreas, and ovary tissues of precocious female *E. sinensis* from different ovary developmental stages to 1) provide a convenient way to identify precocious *E. sinensis* in aquaculture, 2) reveal the tissue-expression profiles in precocious female *E. sinensis* in chronological order, 3) identify potential candidate genes/pathways involved in early ovary development, and 4) provide novel insights into the genetic network of eyestalk and hepatopancreas in regulating ovary development in *E. sinensis*.

## Methods

### Animal sampling and ethics

This study was approved by the Institutional Animal Care and Use Committee (IACUS) of Shanghai Ocean University (Shanghai, China). Sampling procedures complied with the guideline of IACUS on the care and use of animals for scientific purposes. All the *E. sinensis* individuals in this study were collected from the Aquatic Animal Germplasm Station of Shanghai Ocean University (Shanghai, China) and were anesthetized on ice before sampling. In this study, potential precocious female *E. sinensis* individuals were firstly collected according to the ratio of abdominal sternite length (B5-B5’/C5-C5’) and then confirmed by histological observation (Fig. 1).

### Measurement of phenotypic characters and histological observation

For the collected *E. sinensis* individuals, phenotypic characters such as body weight, ovary weight, and hepatopancreas weight were measured, and abdominal sternite length was recorded as previously described [19]. The hepatopancreas index (HSI), gonad index (GI), and ratio of abdominal sternite length were calculated using the following formulas:

Hepatopancreas index = (wet hepatopancreas weight/wet body weight) × 100%,

Gonad index = (Wet gonad weight/wet body weight) × 100%.

Rate of abdominal sternite length = (B5-B5’)/(C5-C5’) (Fig. 1a). Ovary tissues from potential precocious *E. sinensis* individuals were fixed using Bouin’s fixative (Sangon Biotech, China) at room temperature for 24 h. Then, ovary tissue-slices were prepared and stained with hematoxylin-eosin (HE). The tissue-slices were observed under a DM500 microscope system (LEIKA, Germany) and Image Analysis Software Toup View.

### RNA isolation and transcriptome sequencing

According to the histological observation results, precocious *E. sinensis* individuals with ovary developmental stages in major growth stage I, major growth stage II, and sexually mature stage were collected with three biological replicates in each group. Meanwhile, three normally developed two-year-old female *E. sinensis* individuals (sexually mature stage) were also sampled as the control group. The eyestalk, hepatopancreas, and ovary tissues were quickly collected and stored in liquid nitrogen before RNA extraction. Total RNA was extracted from each collected *E. sinensis* individual with RNAiso Reagent (Takara, China) according to the manufacturer’s instructions. RNA integrity and quantity were examined using agarose gel electrophoresis and an Agilent 2100 Bioanalyzer (Agilent, Shanghai, China), respectively. A total of 5 μg RNA with an RNA integrity number (RIN) exceeding 8.0 was used for RNA-seq library construction using the Truseq™ RNA sample Prep Kit for Illumina (Illumina, USA). These indexed libraries were sequenced on an Illumina Hiseq™4000, with 150 bp pair-end reads produced.

### Differential expression and enrichment analysis

After sequencing, raw sequencing reads were first trimmed using Trimmomatic software [20]. Then, clean reads were mapped to our previously assembled reference transcriptome assembly (NCBI TSA accession number: GGQO00000000) using Bowtie 1.0.0 [21]. Gene abundance, the TPM (transcripts per million transcripts) value was measured using the RSEM 1.3.0 software [22]. The resulting data matrix with expression value (TPM) for all the samples was generated and used as input data for differential expression analysis. Then, differentially expressed genes (DEGs) were identified by DESeq2 software using *P* < 0.001 for the false discovery rate (FDR) and a fold change > 2^2^ [23]. After normalizing the DEG TPM values using log2 and mean centered, cluster analysis was performed using the hierarchical cluster method based on the euclidean distance using heatmap module in R. GO and KEGG enrichment analysis of the DEGs was conducted using DAVID annotation software with *P* value < 0.05 [24]. Pearson correlation was calculated and plotted by corrplot package in R.

### qRT-PCR validation

Quantitative real-time PCR (qRT-PCR) was carried out to validate the DEGs identified in this study. Eight DEGs in the ovary, hepatopancreas, and eyestalk were chosen for qRT-PCR assays. PCR primers were designed according to our previous reference transcriptome assembly (Additional file 1: Table S1). In this study, three reference genes *ubiquitin conjugating enzyme (Ube), beta-actin (β-actin),* and *ribosomal S27 fusion protein (S27)* were selected to normalize the gene expression level. qRT-PCR was conducted using SYBR Green Premix Ex Taq (Takara, China) in a QIAxcel real-time PCR system (Qiagen, German). A standard curve was first generated to assess amplification accuracy, and primers with an amplification efficiency between 95 and 105%, and Pearson correlation (R^2^) > 0.98 were chosen for following qRT-PCR experiments. Three biological and three technical replicates were chosen for each selected DEG. The relative expression was estimated using the 2^–ΔΔCt^ method with normally developed, sexually mature *E. sinensis* individuals as a calibration control [25]. Relative expression results were presented as the fold-change relative to normally developed, sexually mature *E. sinensis* individuals. Statistical significance (*P* < 0.05) was determined using one-way ANOVA tests under SPSS 25.0.

## Results

### Phenotypic character measurement and ovary histological observation

On the basis of the ratio of abdominal sternite length and the histological observation from collected potential precocious *E. sinensis* individuals, four groups of *E. sinensis* with different ovary developmental stages were clearly identified. In group I, the ratio was less than 0.7 and no ovary tissue was clearly discovered; in group II, the ratio ranged from 0.71 to 0.85, the ovary developmental stage was in major growth stage I; in group III, the ratio was 0.86 to 1.0, and the ovary developmental stage was in major growth stage II; in group IV, the ratio was greater than 1.0, and the ovary stage was completely mature with clear oocytes (Table 1, Fig. 1b). The hepatopancreas index decreased from 10.45 to 5.40% and the gonad index increased from 0 to 7.59% with the abdominal sternite length ratio increased from 0.70 to 1.00 (Table 1, Fig. 1).

**Table 1:** Information on phenotypic characters and histological observation of precocious *E.sinensis*

Ratio of abdominal sternite length	No. of individuals	Average weight	Average hepatopancreas index	Average gonad index	Ovary developmental stage
< 0.70	6	5.62 ± 0.72 g	10.45 ± 1.56%	0	Not applicable
0.71–0.85	9	13.72 ± 1.23 g	7.16 ± 1.52%	0.16 ± 0.09%	Major growth stage I
0.86–0.99	9	19.47 ± 4.01 g	6.03 ± 1.70%	0.34 ± 0.10%	Major growth stage II
≥1.0	6	40.90 ± 1.68 g	5.40 ± 0.98%	7.59 ± 1.07%	Sexually Mature stage

### Tissue gene expression profiles in precocious *E. sinensis*

**Fig. 2:**
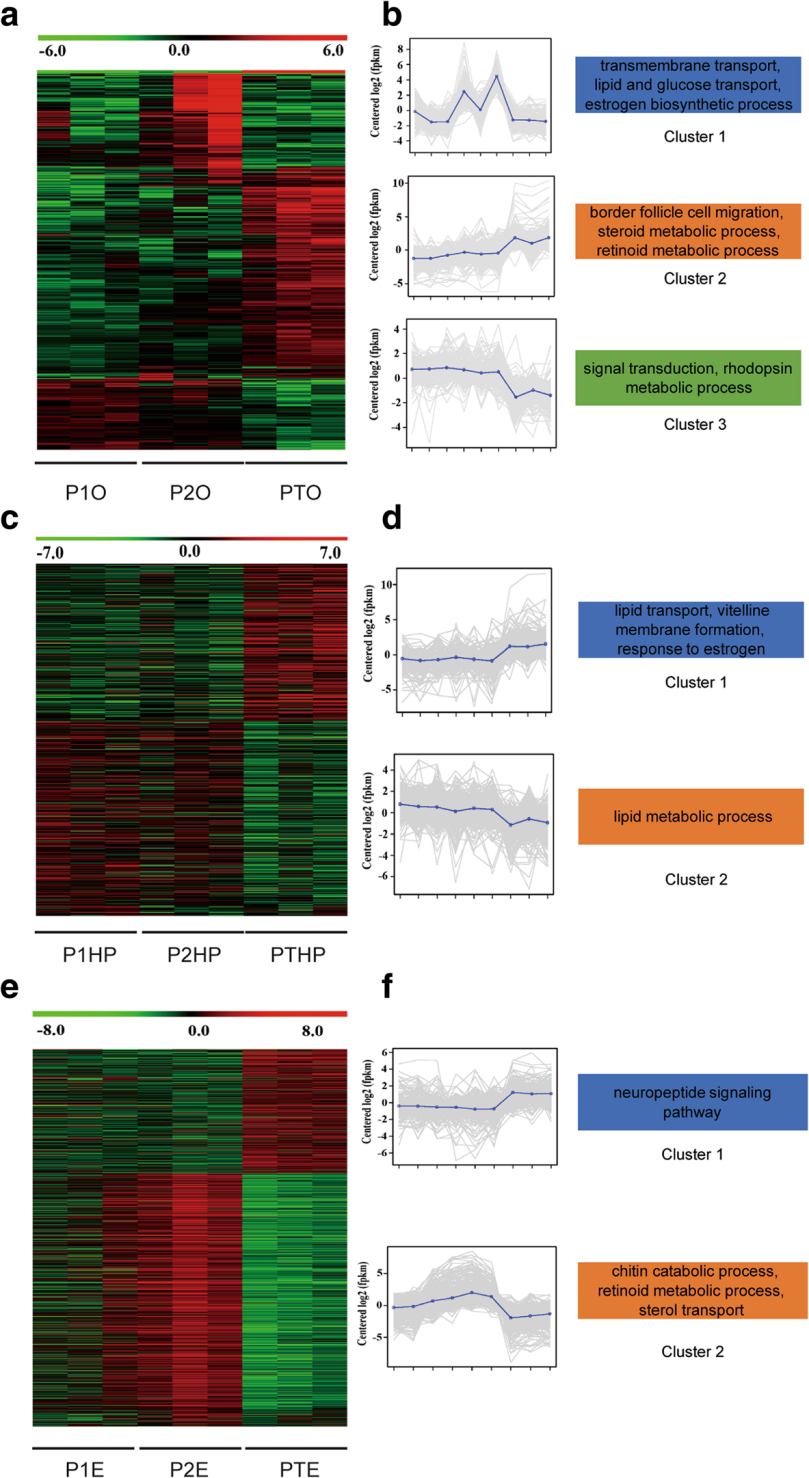
Expression patterns of differentially expressed genes in precocious ovary, hepatopancreas and eyestalk tissues. **a**. Heatmap of differentially expressed genes in the ovary. **b**. Gene expression plot for the three defined clusters. **c**. Heatmap of differentially expressed genes in the hepatopancreas. **d**. Gene expression plot for the two defined clusters. **e**. Heatmap of differentially expressed genes in the eyestalk. **f**. Gene expression plot for the two defined clusters. “P1O”, “P1HP”, and “P1E” indicate precocious ovary, hepatopancreas, and eyestalk with ovary in major growth stage I, respectively; “P2O”, “P2HP”, and “P2E” indicate precocious ovary, hepatopancreas, and eyestalk with ovary in major growth stage II, respectively; “PMO”, “PMHP”, and “PME” indicate precocious ovary, hepatopancreas, and eyestalk with ovary in sexually mature stage, respectively

Regarding ovary tissue, 957 DEGs were identified among different groups of precocious *E. sinensis*. Three clusters were defined based on the hierarchical clustering results revealing different expression patterns in the ovary of precocious *E. sinensis*. In cluster 1, genes such as innexin shaking-B (*shakB*), MFS-type transporter (*SLC18B1*), solute carrier family 13 member 3 (*SLC13A3*), solute carrier family 10 member 6 (*SLC10A6*), low-density lipoprotein receptor 1 (*LDLR-A*), nose resistant to fluoxetine protein 6 (*NRF-6*) genes, and estradiol 17-beta-dehydrogenase 8 (*HSD17B8*) were highly expressed in the major growth stage II group. GO and KEGG enrichment analysis indicated that these genes were enriched in transmembrane transport (GO:0055085), lipid transport (GO:0006869), glucose transport (GO:0015758), estrogen biosynthetic process (GO:0006703), and steroid hormone biosynthesis pathway (cfa00140) (Fig. 2a,b Cluster1, Additional file 2: Table S2). In cluster 2, genes enriched in border follicle cell migration (GO:0007298), retinoid metabolic process (GO:0001523), steroid metabolic process (GO:0008202) and AMPK signaling pathway (hsa04152) were highly expressed in the completely sexually mature precocious group. These genes included myosin heavy chain, non-muscle (*ZIP*), dynamin (*SHI*), ets DNA-binding protein pokkuri (*AOP*), protein catecholamines up (*CATSUP*), very low-density lipoprotein receptor (*VLDLR*), sulfotransferase 1A1 (*SULTLA1*), sortilin-related receptor (*SORL1*), and low-density lipoprotein receptor-related protein (*LRP*) (Fig. 2a,b Cluster 2, Additional file 2: Table S2). Genes in cluster 3, such as receptor-type tyrosine-protein phosphatase kappa (*PTPRK*), neuromedin-B receptor (*NMBR*), NPC intracellular cholesterol transporter 1 (*NPC1*), transient receptor potential protein (*TRP*), eye-specific diacylglycerol kinase (*RDGA*), and D-amino-acid oxidase (*DAO*) were enriched in signal transduction (GO:00071805), rhodopsin metabolic process (GO:0046154), and peroxisome pathway (ssc04146). These genes were highly expressed in major growth stage I (Fig. 2a,b Cluster 3, Additional file 2: Table S2).

**Fig. 3:**
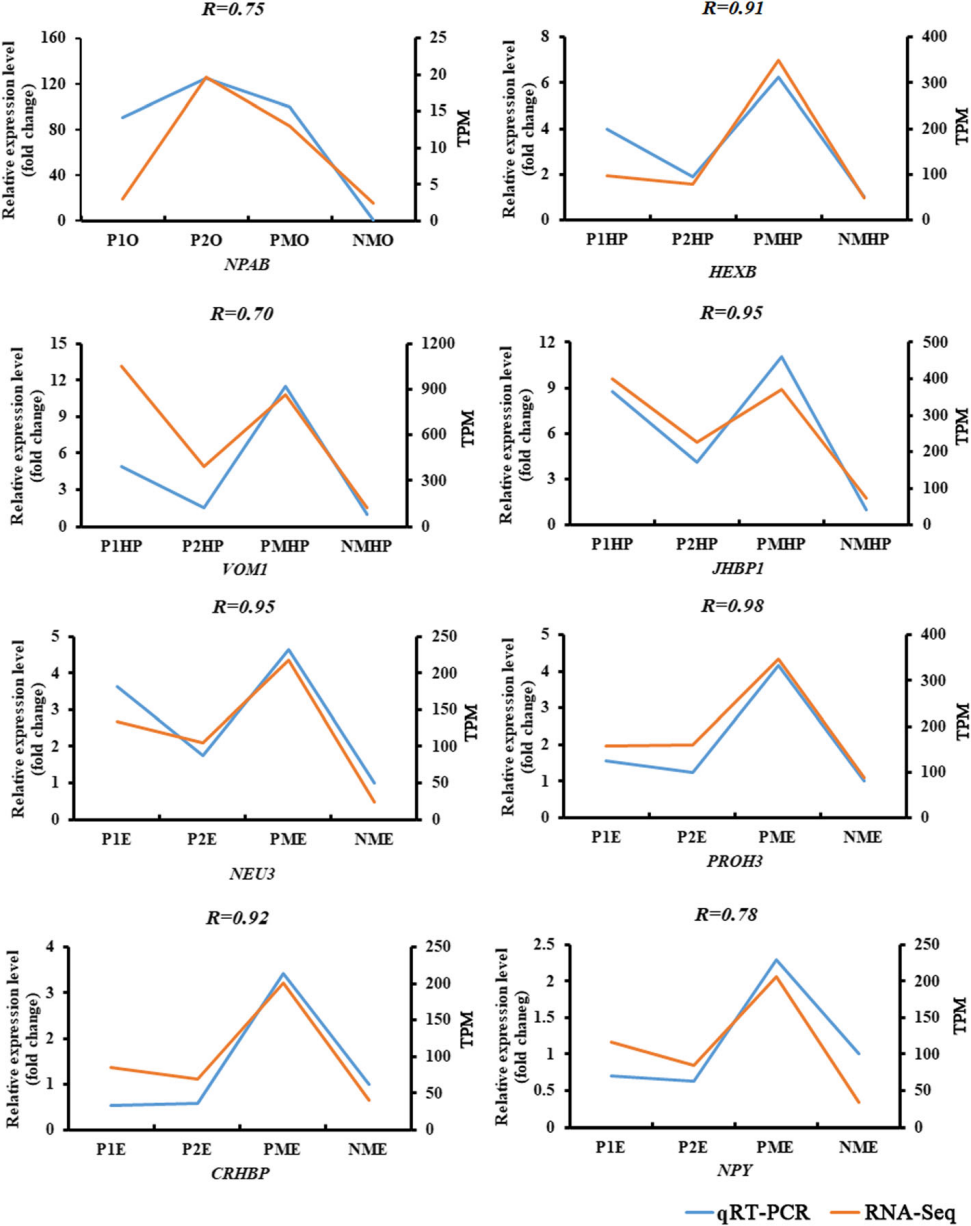
Expression profiles of eight differentially expressed genes from RNA-Seq (orange) and qRT-PCR (blue) at different developmental stages of the ovary “P1O”, “P1HP”, and “P1E” indicate precocious ovary, hepatopancreas, and eyestalk with ovary in major growth stage I, respectively; “P2O”, “P2HP”, and “P2E” indicate precocious ovary, hepatopancreas, and eyestalk with ovary in major growth stage II, respectively; “PMO”, “PMHP”, and “PME” indicate precocious ovary, hepatopancreas, and eyestalk with ovary in sexually mature stage, respectively; "NMO", "NMHP", and "NME" indicate ovary, hepatopancreas, and eyestalk in normal two-year old sexually mature stage

After the comparison of sexually mature precocious ovary with normal two-year-old sexually mature ovary, only 11 DEGs were identified, among which up-regulated genes such as Neuroparsin-A (*NPAB*) and Lipase 3 (*LIP3*) in the precocious ovary were associated with neuropeptide hormone activity and lipid catabolic process (Additional file 3: Table S3, Fig. 3).

A total of 806 DEGs were identified among different groups of precocious hepatopancreas. Two clusters were defined based on the hierarchical clustering results. GO and KEGG enrichment indicated that genes such as sodium/bile acid cotransporter (*SLC10A1*), sodium-dependent nutrient amino acid transporter 1 (*NAAT1*), vitelline membrane outer layer protein 1 (*VMO1*), vitellogenin (*VG*), beta-hexosaminidase subunit beta (*HEXB*), hemolymph juvenile hormone binding protein (*JHBP*), glucosylceramidase (GBA), and arylsulfatase A (ARSA) in cluster 1 were enriched in lipid transport (GO:0006869), vitelline membrane formation (GO:0030704), response to estrogen (GO:0043627) and sphingolipid metabolism pathway (mmu00600) and were highly expressed in the sexually mature precocious group (Fig. 2c,d Cluster 1, Additional file 2: Table S2). Genes such as glycerol-3-phosphate acyltransferase 3 (*GPAT3*), bile salt-activated lipase (*CEL*), lysosomal acid lipase (*LIPA*), and pancreatic triacylglycerol lipase (*PNLIP*) in cluster 2 were associated with lipid metabolic process (GO:0006629) and fat digestion and absorption pathway (mmu04975) (Fig. 2c,d Cluster 2, Additional file 2: Table S2).

After the comparison of normal two-year-old sexually mature hepatopancreas with sexually mature precocious hepatopancreas, 372 DEGs were identified. Genes up-regulated in completely sexually mature precocious hepatopancreas, such as *VMO1* and *VG* were enriched in vitellogenin synthesis, vitelline membrane formation, and lipid transport biological process (Additional file 3: Table S3, Fig. 3).

A total of 1081 DEGs were identified among different groups of precocious eyestalks. Two clusters were defined based on the hierarchical clustering results revealing different expression patterns. GO and KEGG enrichment analysis indicated genes such as neuropeptide F (*NPF*), *MIH, CHH*, vasotocin-neurophysin VT 1 (*VT1*), glycoprotein hormone beta-5 (*GPHB5*), and corticotropin-releasing factor-binding protein (*CRHBP*) in cluster 1 were highly expressed in sexually mature precocious eyestalk and were enriched in neuropeptide signaling pathway (GO:0007218) (Fig. 2e,f Cluster1, Additional file 2: Table S2). Genes such as cuticle protein CP498, cuticle protein AM1159, chitotriosidase-1 (*CHIT1*), probable chitinase 2 (*CHT2*), ATP-binding cassette sub-family G member 5 (*ABCG5*), and ATP-binding cassette sub-family G member 8 (*ABCG8*) in cluster 2 were involved in chitin catabolic process, retinoid metabolic process (GO:0001523), sterol transport (GO:0015918), and amino sugar and nucleotide sugar metabolism pathway (hsa00520) (Fig. 2e,f Cluster 2, Additional file 2: Table S2).

A total of 449 DEGs were identified between normal two-year-old sexually mature eyestalk and sexually mature precocious eyestalk. Up-regulated genes such as *VT1*, *NPF*, prohormone-3 (*PROH3*), *helicostatins*, and pro-neuropeptide Y (*NPY*) in completely sexually mature precocious eyestalk were associated with neuropeptide hormone activity and neuropeptide signaling pathway (Additional file 3: Table S3, Fig. 3).

### DEGs related to neuropeptide hormone activity and lipid transport

Gene expression values from a total of 15 DEGs (GO:0005184, neuropeptide hormone activity) and 12 DEGs (GO:0006869, lipid transport) from the studied individuals were extracted. Out of the 15 DEGs related to neuropeptide hormone activity, 12 were identified in eyestalk tissue, and most of the DEGs (*MIH*, *GPHB5*, *NPA*, *CHH*, *VT1*, *NPF, RPCH*, *PDH1*, *CCAP*, *NPY*) were upregulated in precocious eyestalk than in the normal sexually mature eyestalk. In addition, 11 out of the 12 DEGs related to lipid transport were identified in the hepatopancreas tissue, and most of the DEGs (*SLC10A1*, *apolipophorin*, *VG*, *LDLR-A*, and *SLC10A2*) were upregulated in the precocious hepatopancreas than in the normal sexually mature hepatopancreas (Additional file 4: Table S4).

**Fig. 4:**
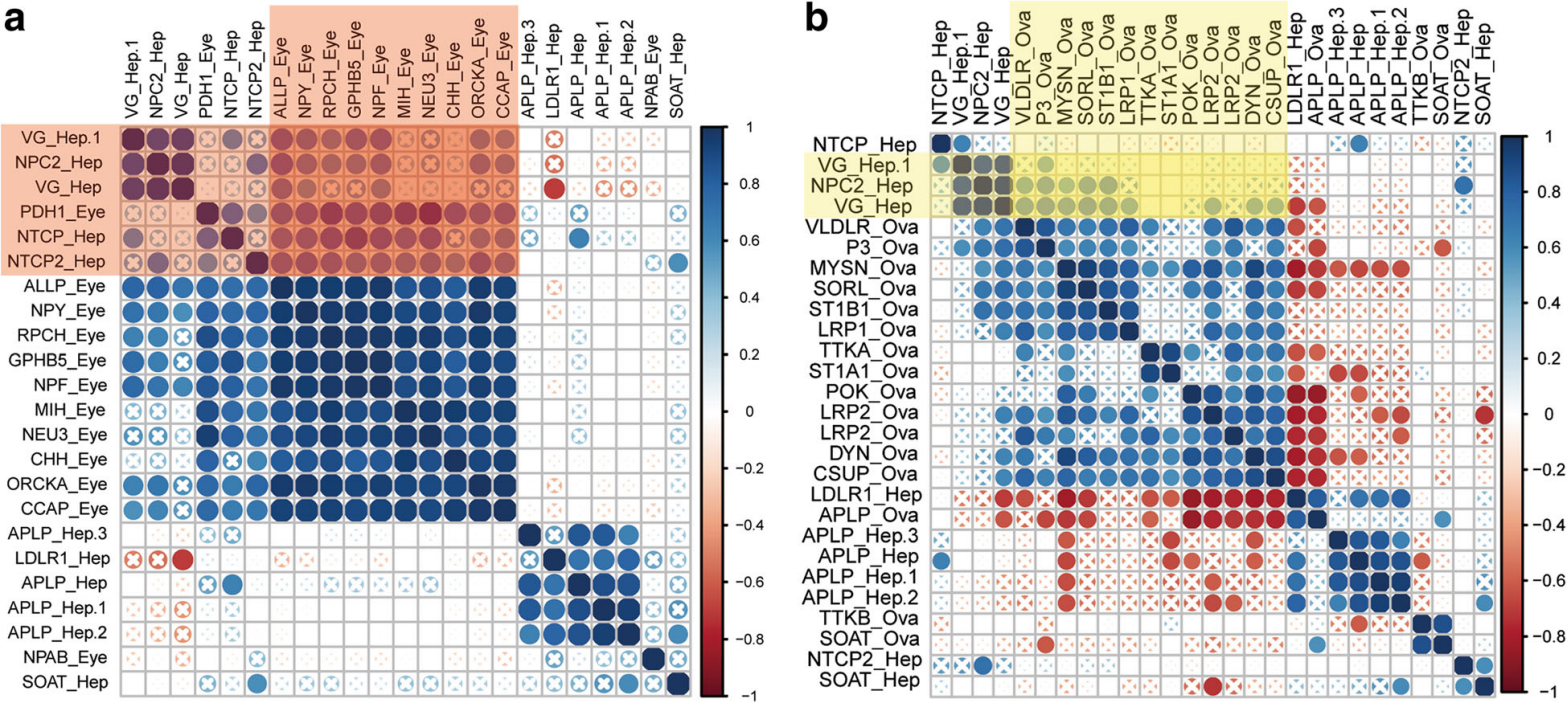
Pearson correlation of gene expression levels of DEGs between tissues (ovary, hepatopancreas, and eyestalk). **a**. Pearson correlation of gene expression levels of DEGs between the hepatopancreas and eyestalk. Red shade indicates positively correlated genes in the hepatopancreas and eyestalk (*P* < 0.05). **b**. Pearson correlation of gene expression levels of DEGs between the hepatopancreas and ovary (*P* < 0.05) “X” symbol indicates *P* value > 0.05 for the Pearson correlation

The correlation coefficient adjacency matrix indicated that the DEGs (*NPY*, *RPCH*, *GHBP5*, *NPF*, *ORCKA*, *CCAP*, *helicostatins*) in the eyestalk annotated as neuropeptide hormone activity positively correlated with the DEGs (*VG*, *NPC2*, *SLC10A1*, *SLC10A2*) in the hepatopancreas annotated as lipid transport (*P* < 0.05) (Fig. 4a, red shade area). Meanwhile, the DEGs (*VG* and *NPC2*) in the hepatopancreas annotated as lipid transport positively correlated with the DEGs in the ovary annotated as oogenesis, border follicle cell migration, steroid metabolic process, and lipid transport (Fig. 4b, yellow shade area) (*P* < 0.05). However, most DEGs in the eyestalk annotated as neuropeptide hormone activity were not correlated with the DEGs in the ovary, and only *SLC10A3* positively correlated with *helicostatins*, *RPCH*, *GHBP5*, and *CCAP* genes (Additional file 5: Figure S1).

## Discussion

Sexual precocity is a complex biological process involving many genes/pathways in specific organs to induce early gonad development [1, 5]. In this study, we utilized the ratio of abdomen sternite length to discriminate ovary developmental stages and believed it is a convenient method for the early detection of potential precocious *E. sinensis*. *E. sinensis* individuals with the ratio of abdomen sternite length above 0.70 during the first year are potential precocious *E. sinensis* that should be abandoned in the aquaculture.

After comparison of the expression profiles of the ovary between two-year-old and one-year-old sexually mature *E. sinensis*, only 11 DEGs were identified and tissue histological observation showed the normal function of precocious ovary. This result indicated that precocious *E. sinensis* individuals are functional and capable of spawning [2]. However, significantly different gene expression profiles were identified in the hepatopancreas and eyestalk between normal and precocious *E. sinensis*, indicating the important function of the hepatopancreas and eyestalk in regulating ovary development.

The hepatopancreas is an essential organ for the energy storage and metabolism of crustaceans, providing the required energy for growth and development [16, 26]. It is also a site for the synthesis and metabolism of certain steroid hormones required by crustaceans during vitellogenesis [17, 27]. In this study, DEGs associated with lipid metabolic process were upregulated in major growth stages I and II during vitellogenesis in precocious *E. sinensis* (Fig. 2d Cluster 2), indicating the initiation of ovary development depends on the lipid metabolism in the hepatopancreas, which may provide energy and steroid hormones for early ovary development [28, 29].

Previous studies also pointed out that nutriments such as sugar/lipid are absorbed and accumulated in the hepatopancreas; and excessive nutrition is transferred to the gonads continuously, thereby inducing early gonad development [30]. Interestingly, DEGs associated with lipid transport were also upregulated in precocious *E. sinensis* in both hepatopancreas and ovary tissues (Fig. 2b Cluster 2, Fig. 3). These up-regulated lipid transport related genes may provide the genetic basis for the lipid transfer from the hepatopancreas to the ovary [29–31]. The hepatopancreas index measured in this study decreased with the increased gonad index, suggesting that the hepatopancreas may transfer the required energy/lipid to the ovary (Fig. 1**)**. Meanwhile, vitellogenin (endogenous and exogenous) is the key factor component in vitellogenesis in *E. sinensis*. Endogenous VG is synthesized by oocyte, while exogenous VG is synthesized in the hepatopancreas and transferred to the ovary [32]. In this study, *VG* gene expression in the hepatopancreas positively correlated with oogenesis, and lipid transport related DEGs in the ovary indicating excessive *VG* expression in the hepatopancreas may stimulate ovary development in precocious *E. sinensis*. *VMO1*, which is associated with vitelline membrane formation, was also up-regulated in precocious *E. sinensis*, indicating that hepatopancreas may also participates in vitelline membrane formation process. Our results confirmed the essential roles of the hepatopancreas in regulating ovary development, energy storage, and steroid hormone synthesis for oogenesis. Vitellogenin synthesis and vitelline membrane formation for vitellogenesis were fulfilled in the hepatopancreas. The intrinsic genetic factors of sexual precocity in *E. sinensis* at some content was caused by abnormal expression of the above-mentioned candidate DEGs in hepatopancreas.

The X-organ-sinus gland complex system in the eyestalk is an important neuroendocrine system in crustaceans [7]. Previous study indicated that regulating the neuroendocrine system of shrimp and crab improve the growth rate and the maturity time, and that eyestalk ablation stimulates gonad development and ovulation [33]. Consistent with previous hypotheses, the present study identified more neuropeptide hormones such as *NPF*, *NPY*, and *prohormone-3* as up-regulated DEGs in the precocious eyestalk in this study. This study indicates the essential regulatory mechanism of the eyestalk in ovary development. *NPF* and *NPY* belonging to the NPY family are neuropeptide hormones that accelerate ovarian maturation in female *Schistocerca gregaria* [34], and regulate visceromotor functions during egg laying [35]. It is well known that *CHH* and *MIH* genes inhibit periodic molting in *E. sinensis*, and *E.sinensis* stop molting and initiate their gonad development after the last reproductive molting during their life [36, 37]. Therefore, extremely highly expressed *CHH* and *MIH* in the eyestalk may inhibit the molting and induce precocity in advance. Interestingly, DEGs in the eyestalk such as *NPY*, *RPCH*, *helicostatins*, *GHBP5*, *NPF*, *ORCKA*, and *CCAP* were positively correlated with *VG* genes expression in the hepatopancreas. This result indicates these neuropeptide hormone genes may target hepatopancreas and induce *VG* expression. However, further functional experiments need to be conducted to confirm the hypothesis (Fig. 4b). All the upregulated genes in the precocious eyestalk indicated that the neuropeptide hormone synthesized in the eyestalk may stimulate ovary development similar to the HPG axis. Our results proved that the eyestalk is indeed an essential organ for gonad development and possibly regulates vitellogenesis in precocious *E. sinensis*. However, the interplay of the eyestalk, hepatopancreas, and ovary requires further functional research.

Sexual precocity is a serious situation in the aquaculture of *E. sinensis*, and scientific researchers and farmers struggled to find solutions and elucidate the genetic mechanism of sexual precocity. In this study, few expression differences were identified between the precocious and normal sexually mature ovary, and early ovary development may be affected by abnormally developed eyestalk and hepatopancreas. Several stimulation factors, such as high temperature, salt, stock density, and nutrition, may induce the metabolic disorder of genes associated with neuropeptide, and steroid hormones, leading to the abundant expression and accumulation of VG in the hepatopancreas and further initiating the ovary development. However, comprehensive functional studies should be conducted to elucidate the genetic mechanism underlying sexual precocity, especially the regulatory mechanism for eyestalk and hepatopancreas. Our study provides valuable genetic resources for the research of sexual precocity in *E. sinensis* in the future. Meanwhile, the effective convenient phenotype measurement method and related candidate DEGs identified in this study provide guidance for the detection of precocious *E. sinensis* in aquaculture.

## Additional files

Additional file 1: **Table S1.** Primers for qPCR confirmation. (DOCX 15 kb)

Additional file 2: **Table S2.** GO and KEGG enrichment analysis for differential expressed genes in ovary, hepatopancreas and eyestalk tissues. (XLSX 21 kb)

Additional file 3: **Table S3.** DEGs between sexually mature precocious and normal sexually mature *E. sinensis*. (XLSX 12 kb)

Additional file 4: **Table S4.** DEGs related to neuropeptide hormone activity and lipid transport identified in this study. (XLSX 12 kb)

Additional file 5: **Figure S1.** Pearson correlation of gene expression levels of DEGs between ovary and eyestalk (*P* < 0.05). “X” symbol indicated *P* value> 0.05 for the Pearson correlation. (TIF 844 kb)

## Abbreviations

DEG: Differentially expressed genes
GI: Gonad index
HE: Hematoxylin-eosin
HPG: Hypothalamus-pituitary-gonad axis
HSI: Hepatopancreas index
RIN: RNA integrity number
TPM: Transcripts per million transcripts
