# Supplementary material for: Tissue expression profiles unveil the gene interaction of hepatopancreas, eyestalk, and ovary in the precocious female Chinese mitten crab, Eriocheir sinensis
Source: BMC Genet. 2019 Jan 25;20:12. doi: 10.1186/s12863-019-0716-1 (PMC6347758; doi:10.1186/s12863-019-0716-1)
Supplement: Supplementary file 1 — Table S1. Primers for qPCR confirmation. (DOCX 15 kb) [file 12863_2019_716_MOESM1_ESM.docx]

Table S1. Primers for qPCR confirmation.

| Primers | Primers sequences (5’-3’) | Product size(bp) | Amplification efficiency |
| --- | --- | --- | --- |
| *NPAB* | F: CAAGGGTCCCGGCGAGTC  R: GAGGCCGCAGGAGCAGAAG | 85 bp | 97% |
| *HEXB* | F: CCGCCAAGATCCACGAGACC  R: GGCGGCTGATGTCAGGGAAA | 126 bp | 105% |
| *VOM1* | F: AGACCGAGGCGTGTCCTGAG  R: GCGTTCACGCTCGTGTTGTC | 94 bp | 101% |
| *JHBP1* | F: GTGTCCCTCGGCATAACGCA  R: GGGAGGCAATGAGGCCATCC | 97 bp | 104% |
| *NEU3* | F: ATGGTGCCCATCAGGTGCAG  R: CGCATTTGGTCTCCGTGCAG | 103 bp | 101% |
| *PROH3* | F: CGCTGAGCTCCGAGTGTGAG  R: TGTTGACGTGGCCCAGACAG | 126 bp | 105% |
| *CRHBP* | F: CTCCCACCCGTCGACGAAAC  R: GCGTCTACCTGCTGAGCCAA | 107 bp | 105% |
| *NPY* | F: GGCAGGCCCAACATATTCAAGAC  R: CCGGCGATAGCGAAGTAGGC | 80 bp | 105% |
| *β-actin* | F: TCATCACCATCGGCAATGA  R: TTGTAAGTGGTCTCGTGGATG | 100 bp | 102% |
| *UBE* | F: TTGCGTTCACAACTCGTATCTACC  R: GTCCGTGAGGAGGGAACAGA | 137 bp | 99% |
| *S27* | F: GGTCGATGACAATGGCAAGA  R: CCACAGTACTGGCGGTCAAA | 105 bp | 105% |
