# Supplementary figures and images for: Tissue expression profiles unveil the gene interaction of hepatopancreas, eyestalk, and ovary in the precocious female Chinese mitten crab, Eriocheir sinensis
Source: BMC Genet. 2019 Jan 25;20:12. doi: 10.1186/s12863-019-0716-1 (PMC6347758; doi:10.1186/s12863-019-0716-1)

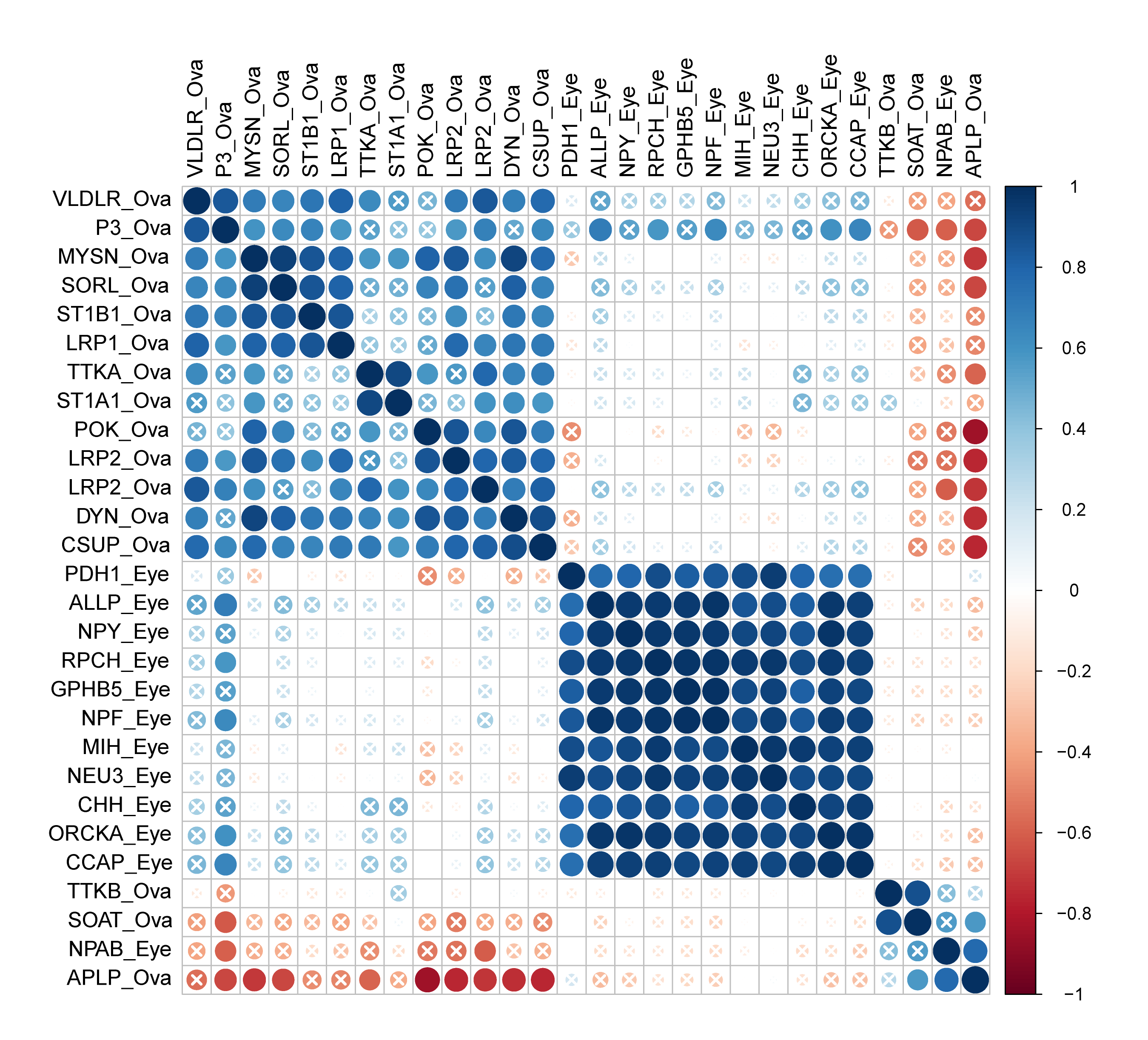

Supplement: Supplementary file 5 — Figure S1. Pearson correlation of gene expression levels of DEGs between ovary and eyestalk (P < 0.05). “X” symbol indicated P value> 0.05 for the Pearson correlation. (TIF 844 kb) [file 12863_2019_716_MOESM5_ESM.tif]
